# Identifying the minimum amplicon sequence depth to adequately predict classes in eDNA-based marine biomonitoring using supervised machine learning

**DOI:** 10.1016/j.csbj.2021.04.005

**Published:** 2021-04-26

**Authors:** Verena Dully, Thomas A. Wilding, Timo Mühlhaus, Thorsten Stoeck

**Affiliations:** a Technische Universität Kaiserslautern, Ecology, D-67663 Kaiserslautern, Germany; b Scottish Association for Marine Science, Scottish Marine Institute, Oban, Scotland, United Kingdom; c Technische Universität Kaiserslautern, Computational Systems Biology, D-67663 Kaiserslautern, Germany

**Keywords:** AMBI, AZTI's marine biotic index, ASV, Amplicon Sequence Variants, AZE, allowable zone of effect, intermediate impact zone, BallWa, ballast water dataset, BasCo, Basque coast dataset, BI, biotic index, bp, base pairs, CE, cage edge, CV, Coefficient of Variance, DADA2, Divisive Amplicon Denoising Algorithm, eDNA, environmental deoxyribonucleic acid, EQ, environmental quality, FM, full model, MDS, multidimensional scaling, microgAMBI, AZTI's marine biotic index based on microbial genes, mtry, numbers of variables tried at each split, n, number, NEB, New England Biolabs, NorSa, Norway salmon dataset, NW, north west, OOB-error, out-of-bag error estimate, PCR, polymerase chain reaction, REF, reference site, RF, random forest algorithm, rRNA, small subunit prokaryotic ribosomal ribonucleic acid, ScoSa, Scottish salmon farm dataset, SML, supervised machine learning, V3-V4, hypervariable gene regions of the 16s rRNA, Biomonitoring, Environmental DNA, Machine learning, Marine, 16S rRNA

## Abstract

Environmental DNA metabarcoding is a powerful approach for use in biomonitoring and impact assessments. Amplicon-based eDNA sequence data are characteristically highly divergent in sequencing depth (total reads per sample) as influenced *inter alia* by the number of samples simultaneously analyzed per sequencing run. The random forest (RF) machine learning algorithm has been successfully employed to accurately classify unknown samples into monitoring categories. To employ RF to eDNA data, and avoid sequencing-depth artifacts, sequence data across samples are normalized using rarefaction, a process that inherently loses information. The aim of this study was to inform future sampling designs in terms of the relationship between sampling depth and RF accuracy. We analyzed three published and one new bacterial amplicon datasets, using a RF, based initially on the maximal rarefied data available (minimum mean of > 30,000 reads across all datasets) to give our baseline performance. We then evaluated the RF classification success based on increasingly rarefied datasets. We found that extreme to moderate rarefaction (50–5000 sequences per sample) was sufficient to achieve prediction performance commensurate to the full data, depending on the classification task. We did not find that the number of classification classes, data balance across classes, or the total number of sequences or samples, were associated with predictive accuracy. We identified the ability of the training data to adequately characterize the classes being mapped as the most important criterion and discuss how this finding can inform future sampling design for eDNA based biomonitoring to reduce costs and computation time.

## Graphical abstract

**Figure:**
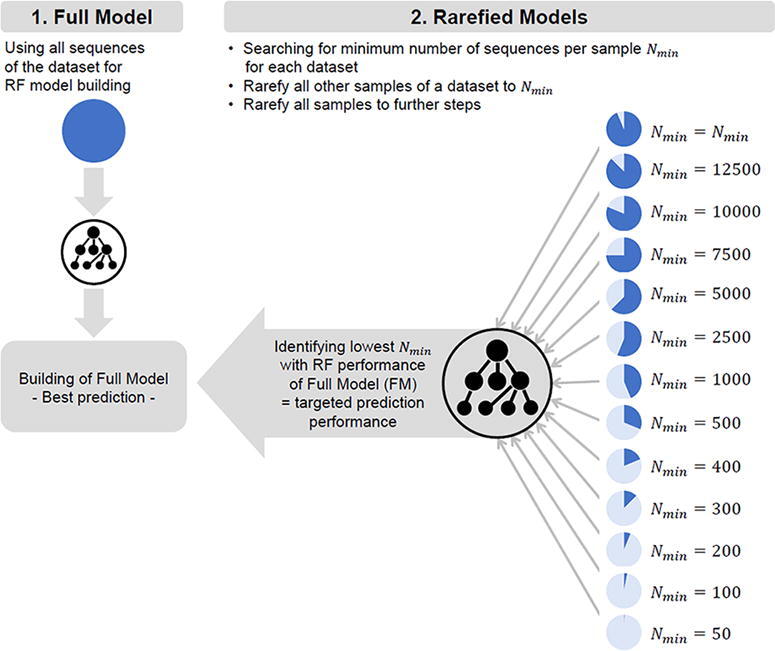

## 1 Introduction

Marine coastal ecosystems offer numerous ecosystem services and therefore are subject to a multitude of stressors from anthropogenic activities, resulting in eutrophication, pollution, overexploitation, and introduction of invasive species [1], [2], [3], [4]. These local stressors are complemented by global effects such as increasing temperatures, sea level rise, and ocean acidification [5], [6]. These stressors may severely affect marine coastal ecosystems and compromise ecosystem services [7]. Therefore, environmental biomonitoring programs for an efficient management and protection of marine coastal ecosystem are in place, which are laid down in national and international Directives, such as the Marine Strategy Framework Directive [8].

The biological component is a backbone of environmental monitoring. In contrast to chemical monitoring approaches, which provide only an environmental quality snapshot, biological indicators are affected by the total range of environmental species they are exposed to, and thus provide a cumulative measure of environmental health [9]. Traditional methods applied to analyze marine bioindicators (mostly meio- and macrofauna) are based on morphological identification and observational surveys. Such surveys are time consuming, expensive, and characterized by low upscaling potential for high-throughput monitoring to resolve environmental changes on small spatial and temporal scales. In addition, evident limitations of this traditional approach are the identification and quantification of rare species and the ability to distinguish morphologically close or identical species (i.e., cryptic species), or poorly characterized juvenile stages of known species [10].

Concerted efforts of the scientific community in recent years were therefore the development of fast, less expensive, and more robust coastal biomonitoring methods with high potential for automation and upscaling. Environmental DNA (eDNA) metabarcoding of marine communities emerged as a very promising strategy that meets these requirements. It uses short, standardized gene regions obtained from environmental samples as internal taxon tags to provide rapid characterization of whole communities. Recently, a remarkable number of applied environmental metabarcoding studies tested the potential use of metabarcoding data to assess the ecological status of natural marine communities exposed to various anthropogenic pressures (reviewed in [11]). In specific, bacteria and protists, which dominate most ecosystems in terms of biomass, and structural and functional diversity are likely the best option on which to perform efficient next-generation marine biomonitoring [9], [12], [13], [14], [15], [16], [17], [18], [19], [20], [21], [22].

A major challenge in eDNA-based biomonitoring was the inference of biotic indices (BI), which inform about environmental quality (EQ) of the ecosystem under study. One solution was the development of specific indices such as the microgAMBI that exploits the taxonomic information obtained from eDNA metabarcodes and the ecological function of identified microbial taxa [12], [13]. Because of severely incomplete gene reference databases for microbial taxa, the microgAMBI relies on the ecological functions of higher taxon ranks rather than species, which were obtained from previously published reports. Consequently, a large proportion of the obtained eDNA metabarcode datasets, which could not be assigned to the required taxonomic ranks, but which may be important indicators, cannot be used for the inference of the microgAMBI. As an alternative, other authors correlated obtained amplicon sequence variants (ASVs) of microbial communities to gradients of environmental stressors and assigned an index value to significantly correlating ASVs [16], [17]. These indicator ASVs were then used as parameters in modified versions of traditional BIs originally developed for macroinvertebrate bioindicators. The most promising approach, however, to infer EQ from metabarcode datasets is supervised machine learning (SML) [16], [23]. The principle and power of this approach is reviewed in detail in Cordier et al. 2018 [9]. In brief, SML is taxonomy-independent and does not rely on available knowledge about the ecology of the species hidden behind microbial ASVs. This eliminates difficulties relating to incomplete nucleotide reference databases and a lack of knowledge about the ecology of numerous yet unknown marine microbes. Classification via SML is first used on a training dataset, which consists of two sets of data that are obtained from the same samples. These are the ASVs of the microbial community in this sample and the reference labels (for example the BI obtained from conventional macroinvertebrate monitoring of the same sample). A predictive model is trained to link specific bacterial ASVs to specific reference groups. The accuracy of a model can then be evaluated with a *kappa* statistic: kappa values ranging from 0.01 to 0.2 indicate “poor agreement” between two classifications (traditional macrofauna-based vs. eDNA-based EQ classification); and values of > 0.8 indicate “perfect agreement” [24]. The successfully trained model can then be used for making predictions of reference labels on upcoming genetic metabarcode samples without collecting additional data as reference. The most successfully applied SML approach for classification using ASVs is Random Forest (RF) for massive and noisy DNA amplicon datasets [9], [25], [26], [27], [28].

This approach marrying environmental genomics and BI inference is of high relevance for industry and politics (environmental management and decision making) alike. A decisive criterion for the implementation of this approach in routine monitoring practice is the costs associated with each technology. A part of this costs depends on the required depths of sequencing to make as accurate as possible inference of BI and EQ for an ecosystem under surveillance. In our study we use three eDNA datasets from previous reports and one new original dataset to infer the minimal sequence depths for marine microbial communities to exploit these data with an RF approach to infer the origin of ballast water and to predict EQ of ecosystems under the impact of urban infrastructure and of aquaculture disturbance. The main questions are: (1) What is the lower limit of sequences for accurate RF predictions in marine coastal monitoring using microbial communities? (2) Is this limit the same for different monitoring targets? A major goal of the study is to inform adequate sampling designs for future eDNA metabarcoding-based marine coastal monitoring surveys.

## 2 Methods

### 2.1 Datasets

We have analyzed four datasets of bacterial Illumina amplicons of the hypervariable V3-V4 16S rRNA gene region. The first dataset [29] included 6,213,619 sequences, obtained from 51 sediment samples of the Basque coast, subjected to various anthropogenic impacts [12]. The authors inferred a novel biotic index, microgAMBI, from these data to assess EQ for each of the samples. The second dataset [30], published by Gerhard and Gunsch [31] included 22,105,927 sequences, obtained from 68 ballast and harbor water samples, to train a Random Forest algorithm for the prediction of geographic ballast water origin. The third dataset [32] included 15,135,391 sequences, obtained from 129 sediment samples to predict the biotic index AMBI for the assessment of aquaculture-induced benthic disturbance at five Norwegian open cage salmon (*Salmo salar*) farming sites [9]. The fourth dataset [33] is original and was obtained from a time series of a Scottish salmon (*Salmo salar*) farm to predict distance from farm and the salmon production phase in which samples were collected. This dataset included 9,496,674 sequences in 76 samples. Details about sampling and data acquisition for this dataset will be described in the following section.

### 2.2 Sampling of Scottish salmon farm sediment

Sediment was collected at three stations along a northwest (NW) transect from the northwesterly cage edge (CE) to a reference site (REF) in the direction of the prevailing current flow, located ca. 800 m distant from the CE. An intermediate impact zone (AZE) was located at ca. 100 m distance from the cage edge. Sampling occurred monthly from March 2018 to March 2019. Due to weather conditions, sampling could not be conducted in November and December 2018. At each site, three biological replicates were taken from a van Veen grab (0.1 m^2^ area), each replicate consisting of 10 g of surface sediment (upper few millimeters) collected using plastic spatulas. Immediately following collection, samples were stored in the dark and on ice (max. 6 h) and then stored at −20 °C. For the purposes of shipping, samples were then defrosted overnight (4 °C) then transferred to equal volumes of LifeGuard nucleic acid preservation solution (Qiagen) until further processing for eDNA metabarcoding.

### 2.3 DNA extraction, amplification and Illumina sequencing of Scottish salmon farm samples

Following our previously described protocol [16], environmental DNA was obtained from sediment samples using the PowerSoil DNA kit (Qiagen, Hilden, Germany) according to the manufacturer’s manual. As DNA metabarcodes, we obtained the ca. 450 bp long hypervariable V3-V4 region of the bacterial 16S rRNA gene. The PCR protocol with the Bakt_341F (CCTACGGGNGGCWGCAG) and the Bakt_805R (GACTACHVGGGTATCTAATCC) primer pair [34] employed an initial activation step of NEB’s Phusion High-Fidelity DNA polymerase at 98 °C for 30 s, followed by 27 identical three-step cycles consisting of 98 °C for 10 s, 62 °C for 30 s, and 72 °C for 30 s; then a final 5-min extension at 72 °C as previously described [16]. Standard negative controls were run with each PCR assay using the same reaction mixture as described above without adding template DNA to the mixture. From the resulting PCR products, sequencing libraries were constructed using the NEB Next® Ultra™ DNA Library Prep Kit for Illumina (NEB, USA). The quality of the libraries was assessed with an Agilent Bioanalyzer 2100 system. V3-V4 libraries were sequenced on an Illumina MiSeq platform, generating 2x300-bp paired-end reads. A standard negative control of a DNA template-free library as well as with PhiX Control v3 library spiked in was run with the samples.

### 2.4 Sequence data processing for all four datasets

Sequences were processed using the Divisive Amplicon Denoising Algorithm DADA2 [35], as described for hypervariable taxonomic marker genes from metabarcoding studies [15] with the model trained on Illumina runs and the following criteria: bacterial V3-V4 sequences were filtered using *filterAndTrim* according to the instructions with truncLen = 225 for V3-V4 sequences and trunclLen = 150 for V4 sequences. To maximize the quality of the final sequence reads used for downstream analyses, we chose the following maxEE values for the individual data set: BasCo = 1, BallWa = 1, NorSa = 2, ScoSa = 1. Bacterial sequences were merged using 20 base pairs overlap with allowed mismatch of 2. To minimize ecologically uninformative noise, only ASVs with at least 50 reads were maintained for downstream analyses, similar to previous publications [9], [36], [37]. Samples with less than 15,000 reads were discarded. Furthermore, the South African harbor and ballast water samples from the BallWa dataset consisted of only eight samples. This is a too small sample size to allow location-specific discrimination, and, therefore, we eliminated these eight samples. Thus, fewer samples were used for our analyses than were included in the original data sets. In summary, for our analyses we included 39 samples for the Basque costal dataset (BasCo), 59 samples for the ballast water dataset (BallWa), 95 samples for the Norwegian salmon farm dataset (NorSa) and 76 samples for the new Scottish salmon farm dataset (ScoSa). After processing, the ScoSa dataset was split near-equal (in terms of sample numbers) to represent salmon production phases. These production phases were defined as pre-production phase (n samples = 25, collected between March and May 2018), early salmon production phase (n samples = 24, collected between June and August 2018) and late salmon production phase (n samples = 27, collected between September 2018 and March 2019). For details, we refer to Supplementary File 1. Sampling in May 2018 occurred immediately after addition of salmon breed to the cages. Thus, in the pre-production phase, no salmon-related impact on the seafloor is expected. In the early salmon production phase, the average fish biomass was 107 tons in the aquaculture installation under study, whereas in the late production phase, this number had increased to on average of 680 tons.

### 2.5 Supervised Machine learning (SML) predictions

Using RF, we predicted the following measures for the four different datasets: The microgAMBI class (“high”, “good”, “moderate” or “poor”) was predicted for the BasCo dataset, using the microgAMBI identified for each sample in the original publication [12]. Geographic origin (Singapore, USA or China, excluding South Africa) of ship ballast water was predicted for the BallWa dataset, using the ground truth data for each sample from the original publication [31]. The AMBI biotic index class (“good”, “moderate”, “poor” or “bad”) was predicted for the NorSa dataset, which was obtained as reference from an official compliance monitoring survey of these farms using benthic macroinvertebrates [9]. Additionally, the farm of sample origin was predicted. For the new ScoSa dataset, we also predicted two variables for each sample, namely distance from farm and salmon production phase in which samples were collected. Predictive models were trained using the RF algorithm [38] implemented in the randomForest v. 4.6.14 package for classification and regression [39]. First, RF is used on a training dataset, one for each of the four bacterial ASV datasets used in this study (BasCo, BallWa, NorSa and ScoSa). Such training data consist of two sets of data that are obtained from the same sample: (a) the obtained bacterial ASVs as features and (b) the reference labels. These reference labels were microgAMBI class, ballast water origin, AMBI class/Farm and distance/salmon production phase for the BasCo, BallWa, NorSa and ScoSa datasets, respectively. The RF algorithm is then trained to relate specific (combinations of) ASVs to defined reference label values (regression) or categories (classification).

An essential feature of the RF algorithm is its use of out-of-bag (OOB) samples. For each observation, a random forest predictor is constructed by averaging only those decision trees in which this observation did not appear. Therefore, an OOB error estimate (OOB-E) is almost identical to that obtained by N-fold cross-validation [40]. Setting the RF parameters for classification approaches, the inventors recommend determining the default *mtry* value to the square root number of features.

In the first step, so-called “full models” (FM) were calculated exploiting all available sequences of each of the four datasets. Prior to RF, we transformed the ASV-to-sample matrix into a relative abundance matrix for each ASV (using the number of reads for the respective ASV divided by the number of all reads in a sample) to compensate for any differences in the sequencing depth between samples [12], [31]. With this matrix RF models were calculated using features and reference labels as mentioned above. For each model we ran 6000 trees. Randomly chosen datasets did not show any OOB-E improvements when increasing the number of decision trees further. For each dataset we repeated the calculation of the FM was repeated several times: for the first FM, the *mtry* value was set to default as recommended. Depending on this default value, six further models were calculated, each with the default *mtry* value plus/minus one, plus/minus two and plus/minus three. Each of these analyses were repeated two times using different base trees to secure the prediction capacity of the full model. The R package *caret* [41] was used to infer *kappa* statistics for each RF model.

**Fig. 1:**
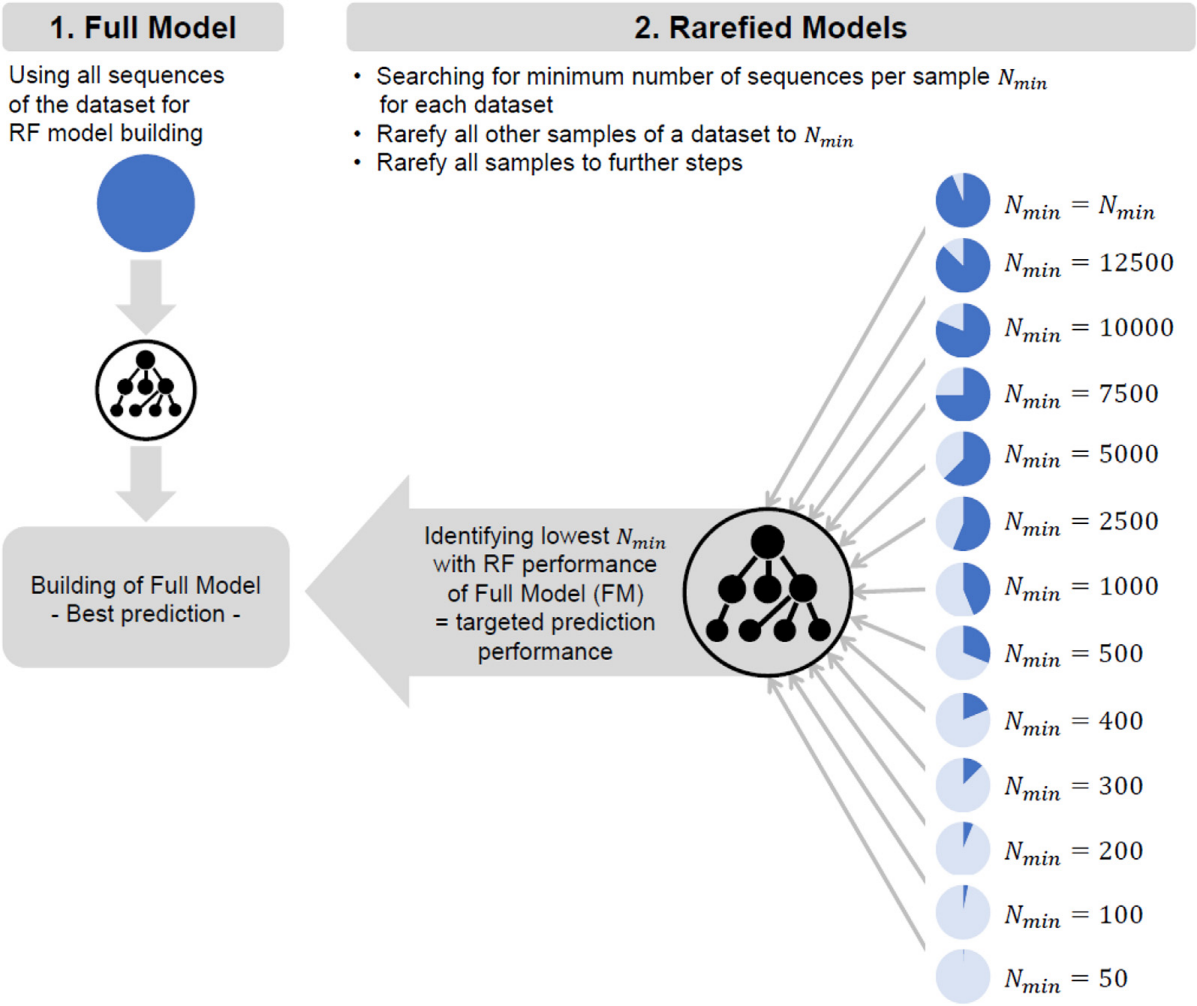
Workflow. Using the full ASVs-to-sample matrix from each dataset analyzed in this study, we build RF models for each of these datasets. The best model of each dataset (=full model) was then used a reference (benchmark) to assess to which degree sequences can be removed from each dataset (=downsampling) without losing prediction performance compared to the full model. The full model is thus the targeted RF prediction performance.

In the second step we then constructed RF models with the rarefied (downsampled) datasets to ask the following question: what minimum number of sequences within a sample is required to obtain an RF prediction performance which is of the same or similar quality as the prediction obtained from the full dataset (FM analysis). From here on, we refer to the prediction accuracy obtained from the full dataset (FM) as “targeted RF prediction performance”. From each of the four full datasets, we created 13 rarefied datasets (52 datasets in total). With exception of the BallWa dataset, the number of sequences per sample in the first rarefied dataset was equal to the number of sequences obtained for the sample with the lowest sequence coverage. This was 15,177 for the ScoSa dataset, 16,501 for the BasCo dataset, and 15,048 for the NorSa dataset. Because the lowest number of sequences per sample within the BallWa dataset was nearly twice as high as for the other three datasets, we have set the number of this first downsampled BallWa model to 15,000 sequences to enable more solid comparisons with the other three datasets. Downsampling for the following 12 models in each of the four datasets employed 12,500, 10,000, 7500, 5000, 2500, 1000, 500, 400, 300, 200, 100 and 50 sequences. For each of the 52 downsampled datasets, RF analyses were conducted as described above for the full model. A schematic graphic of this study design is shown in Fig. 1. Additionally, exemplary information about model construction and downsampling can be found in Supplementary File 2.

## 3 Results

### 3.1 Sequence data overview and rarefaction

**Fig. 2:**
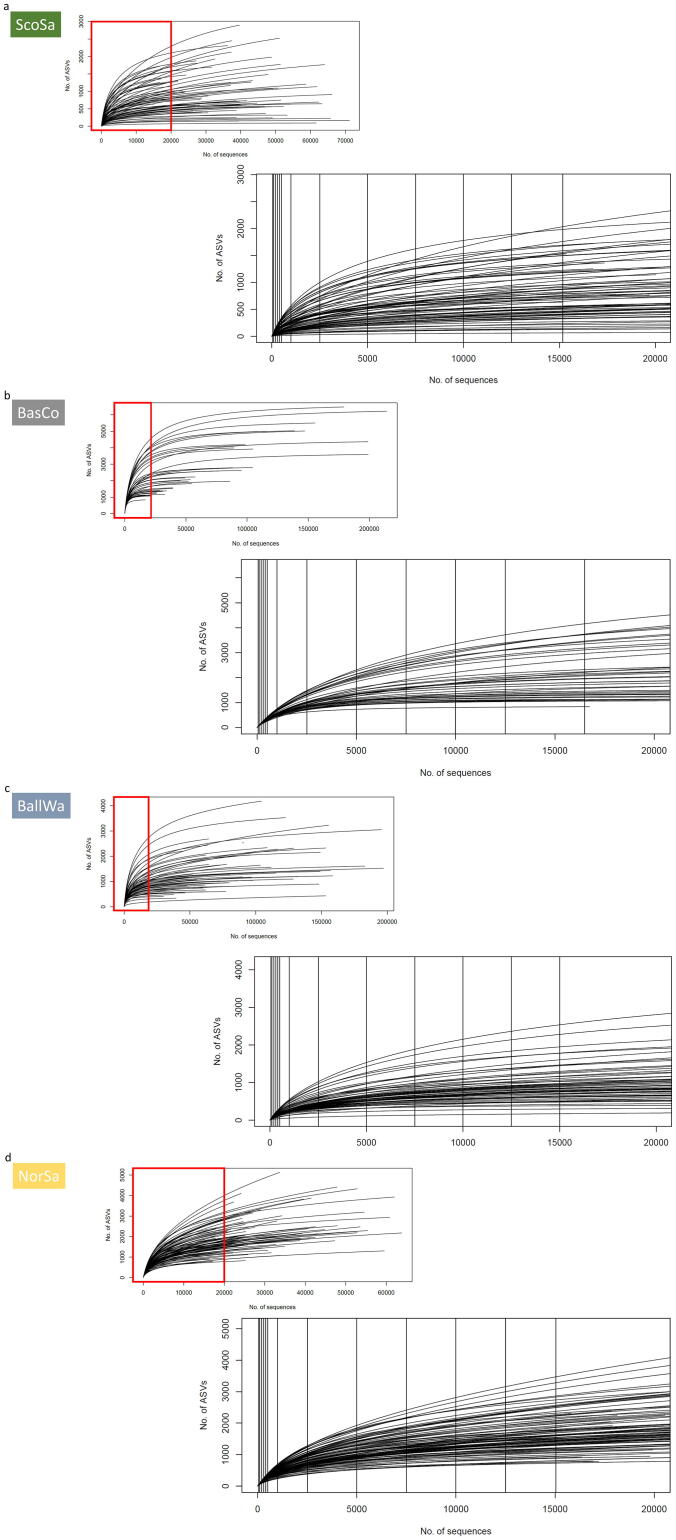
Rarefaction curves (sampling saturation profiles) of the datasets used in this study. (a) ScoSa (Scottish salmon farm sediment samples) dataset; (b) BasCo (marine sediment samples from the Basque coast); (c) BallWa (ballast water samples); (d) NorSa (Norwegian salmon farm sediment samples). The upper smaller graphics shows the full data of each dataset, based on which the full model used to define the “targeted RF prediction performance” was build (see Fig. 1). The red square is the excerpt of the full dataset that is displayed in the lower graphics. In the lower graphics, sampling sizes used for downsampling (see Fig. 1) are marked with vertical lines. This visualizes the level of sample saturation (ASV coverage) at each downsampling size for each of the four datasets. (For interpretation of the references to colour in this figure legend, the reader is referred to the web version of this article.)

**Fig. 3:**
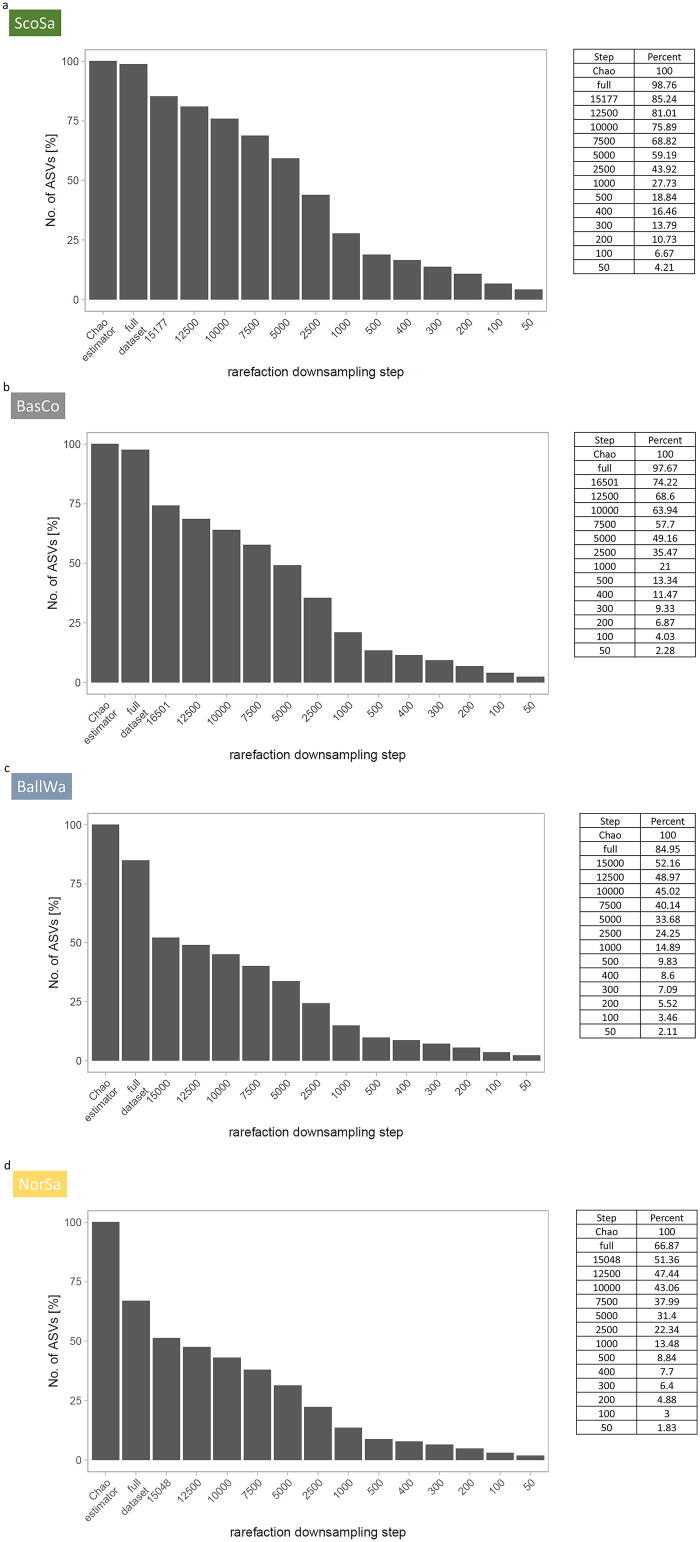
Relative proportion of remaining ASVs at individual downsampling steps for the ScoSa dataset (a), the BasCo dataset (b), the BallWa dataset (c) and the NorSa dataset (d). The Chao1 estimator was used to infer the maximum number of ASVs from each dataset, which is the number of ASVs that could be detected in theory, when all ASVs were sampled in each dataset. This number was set as 100%. “Full” refers to the actually sampled ASVs in all datasets and indicates the discrepancy between the actual ASVs in a dataset and the expected number of ASVs if they were sampled to completion (=Chao1 value).

The number of merged high-quality bacterial amplicons and obtained ASVs for the individual datasets were as follows (amplicons/ASVs): ScoSa 2,653,449/28,151, BasCo 2,310,195/63,943, BallWa 5,024,841/49,843, and NorSa 2,472,237/89,883. The number of ASVs with at least 50 sequence reads, which were used for downstream RF analyses were 3039 (ScoSa), 8406 (BasCo), 6318 (BallWa) and 6012 (NorSa). At their maximum sampling depth, nearly all samples were approaching sample saturation (Fig. 2a–d). Compared to the Chao1 estimator (=100% ASV coverage), the full sequence datasets reached a coverage of 99% (ScoSa), 98% (BasCo), 85% (BallWa) and 67% (NorSa) (Fig. 3a–d). The decrease of coverage (saturation) with downsampling for subsequent RF analyses was notably different for the four datasets. As an example, when downsampling each dataset to 5000 reads, the coverage was 59% for the ScoSa dataset, 49% for the BasCo dataset, 34% for the BallWa dataset and 31% for the NorSa dataset. At the lowest sequence number (n sequences = 50), all samples were severely undersampled with saturation ranging between 4.2% (ScoSa, Figs. 2a and 3a) and 1.8% (NorSa, Figs. 2d and 3d) compared to the full community ASV richness as estimated by Chao1.

### 3.2 Random forest predictions of full and downsampled datasets

**Fig. 4:**
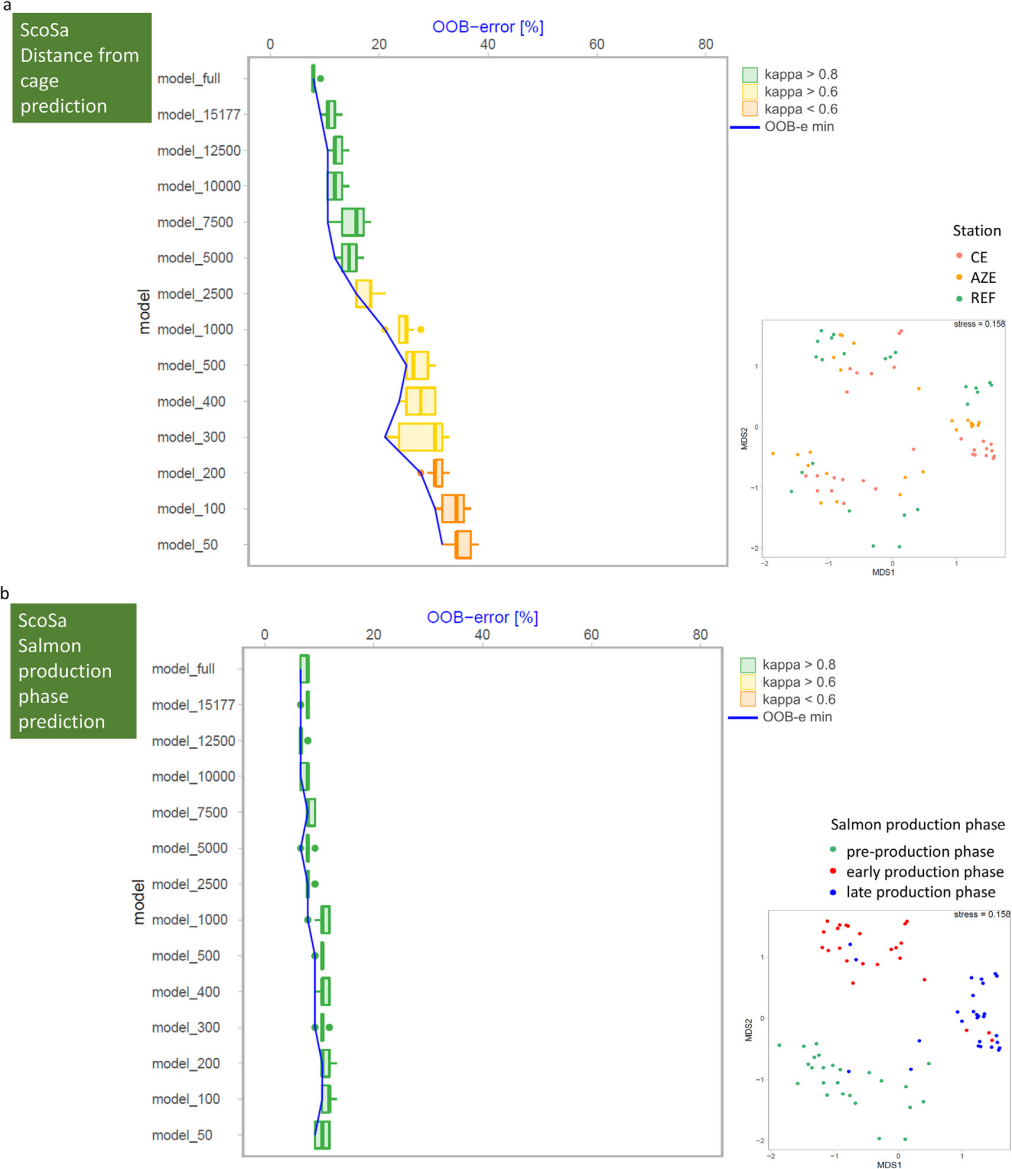
Change of OOB (out of bag error) and kappa value with decreasing dataset size (number of sequences) used for RF prediction for the ScoSa dataset to predict distance from the salmon cage site (a), and the salmon production phase (b) based on benthic bacterial community composition. The boxplot of each downsampled dataset consists of RF prediction results obtained from 21 models. Each boxplot shows median, quartiles (25%–75%), min and max values as well as outliers. Kappa values of > 0.8 (marked in green) indicate “perfect agreement” between observed and predicted classifications (distance from salmon cages for the ScoSa distance dataset in Fig. 4a and salmon production phase in Fig. 4b). Kappa values marked in red (<0.6) indicate poor agreement. In case of distance prediction (Fig. 4a), perfect agreements can be achieved when the full dataset is downsampled to 5000 sequences. In case of salmon production phase predictions as few as 50 sequences still allow for a perfect prediction accuracy compared to the full model (Fig. 4b). Nonmetric multidimensional scaling (NMDS) plots in the lower right corner show the clustering of all individual samples of the full ASV dataset, colored by the specific prediction classes. (For interpretation of the references to colour in this figure legend, the reader is referred to the web version of this article.)

ScoSa (Fig. 4): For the ScoSa dataset we predicted the distance of samples from the salmon farm and the salmon production phase based on the V3-V4 metabarcodes of the benthic bacterial communities. To predict the distance from the salmon farm (Fig. 4a, prediction categories: cage edge, allowable zone of effect, reference), the RF model, which was trained on the full dataset, achieved a mean prediction accuracy of 89.2% (mean out-of-bag error: 10.8%) at a kappa > 0.8. When downsampling, kappa remained above this threshold for “almost perfect agreement” down to 5000 sequences per sample. At this sampling size, the mean prediction accuracy was still 82.2%. Thus, a minimum of 5000 sequences within each sample was required to achieve the targeted RF prediction performance (FM-based prediction accuracy as reference). At sampling sizes ranging between 2500 and 300 sequences per sample, RF could still predict the distance from the salmon farm with a minimal precision of 71.3% (model_300) at a kappa ranging between 0.6 and 0.8 (moderate agreement). Poor agreement was obtained when less than 300 sequences per sample were used for the RF model.

When predicting the salmon production phase in which a specific sample was taken (Fig. 4b, prediction categories: pre-production phase, early production phase, late production phase), prediction accuracy was 92.6% for the full dataset at a kappa of 0.88. When downsampling the full ScoSa dataset, 50 sequences per sample emerged as sufficient to maintain a prediction accuracy as high as 89.5% at an almost perfect agreement (kappa > 0.8 (0.82)) between the predicted and the actual salmon production phase of a sample. Thus, as few as 50 sequences within each sample were sufficient to achieve the targeted RF prediction performance.

**Fig. 5:**
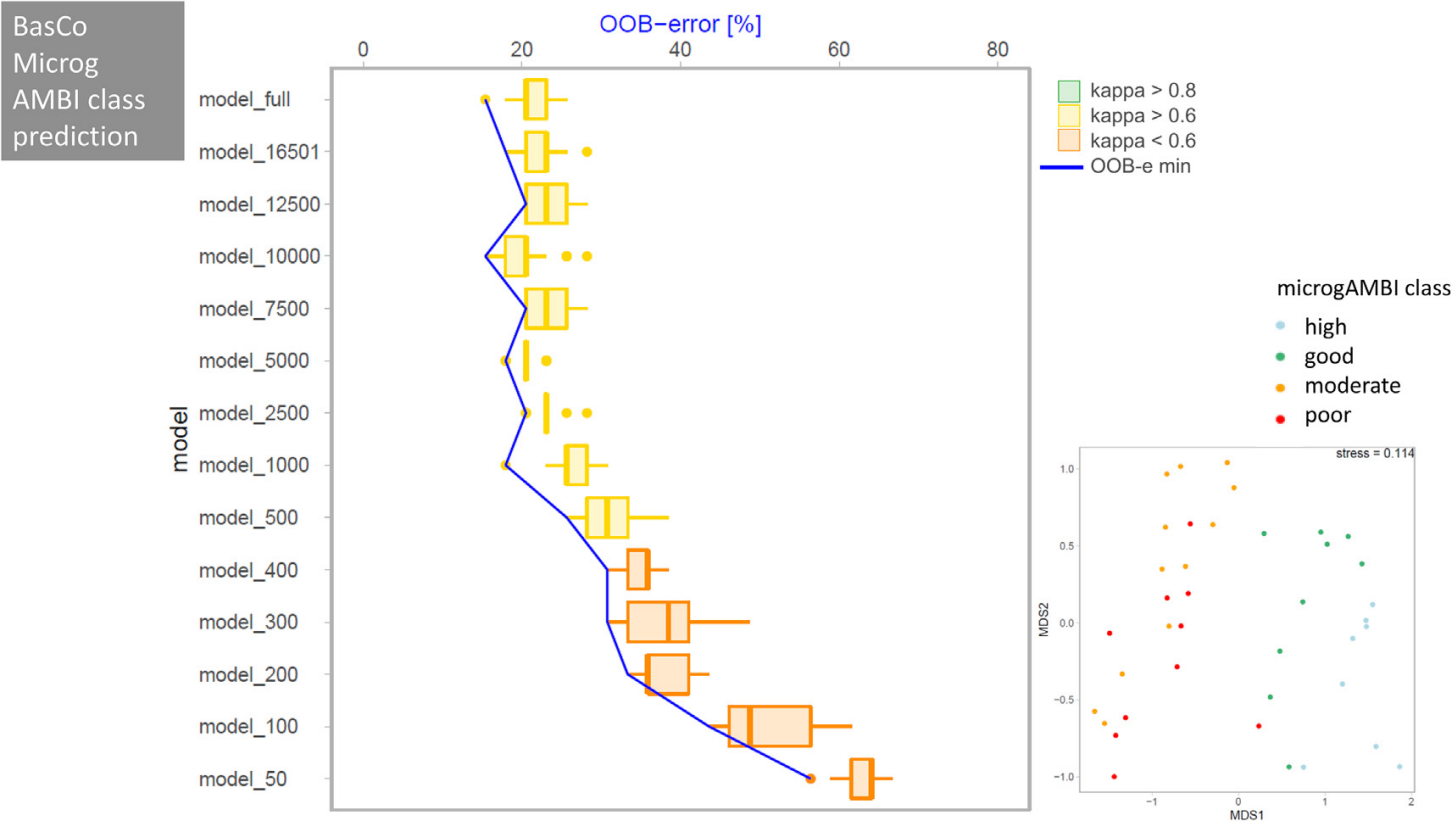
Change of OOB (out of bag error) and kappa value with decreasing dataset size (number of sequences) used for RF prediction for the BasCo to predict microgAMBI index based on benthic bacterial community composition. For further details see legend of Fig. 4.

BasCo (Fig. 5): For the BasCo dataset we predicted the biotic index microgAMBI, based on which the ecological quality (EQ) of a sample was inferred (prediction categories: high, good, moderate, or poor ecological quality). Even when the full dataset was used to train the RF model, the mean precision of prediction was only 78.3% at moderate agreement (kappa = 0.71) between reference EQ values and predicted EQ values. To achieve the targeted RF prediction performance of the full model(based on 214,250 sequences per sample) a minimum of 5000 sequences per sample was sufficient. Kappa values indicated moderate agreement down to 500 sequences per sample. Compared to the full dataset, the precision decreased, however, for 12.5% (OOB error at model_500: 31.5%).

**Fig. 6:**
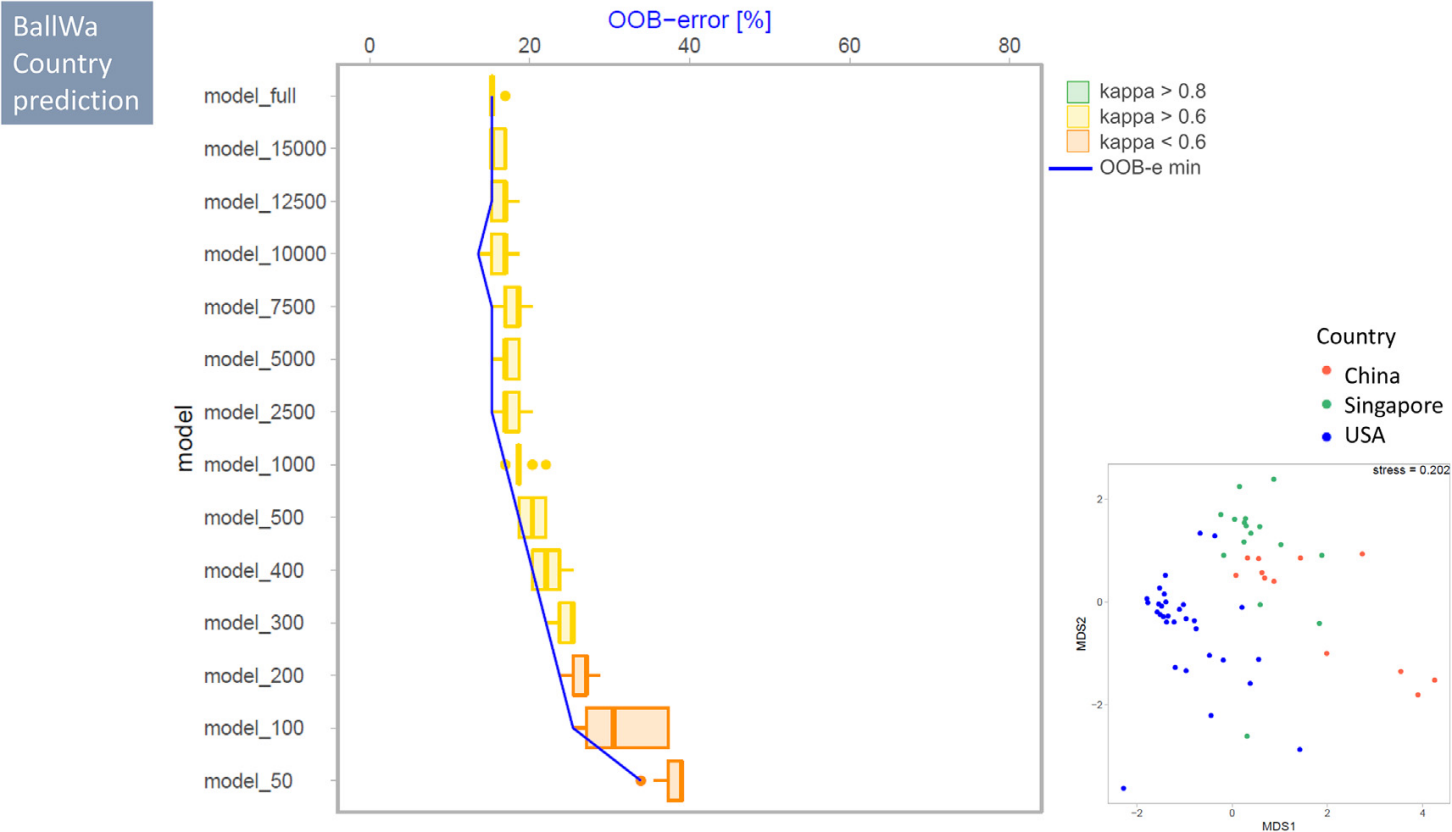
Change of OOB (out of bag error) and kappa value with decreasing dataset size (number of sequences) used for RF prediction for the BallWa dataset to predict country of origin for ballast water samples based on bacterial community composition. For further details see legend of Fig. 4.

BallWa (Fig. 6): For the BallWa dataset we predicted the origin of ballast water samples using the bacterial V3-V4 16S rDNA markers detected in each of the 59 individual samples (categories: China, Singapore, USA). Despite this dataset had the highest number of sequences per sample of all four datasets tested, kappa statistics returned only a moderate agreement between the actual origin of a ballast water sample and the predicted origin even for the full dataset. Mean precision for origin prediction for the full dataset was 84.6% (kappa = 0.74). In the downsampled datasets, at least 2500 sequences per sample were required to match the performance category of the full dataset (targeted RF prediction performance). At this number of sequences within each sample, the mean prediction accuracy remained in the same order of magnitude compared to the full dataset (82.2% for model_2500) at moderate agreement (kappa = 0.70). Down to 300 sequences per sample, kappa remained in this category with a mean prediction accuracy of 75.3%. Only when less than 300 sequences per sample were used, agreement between observed and predicted sample origin was poor with OOB errors exceeding 25%.

**Fig. 7:**
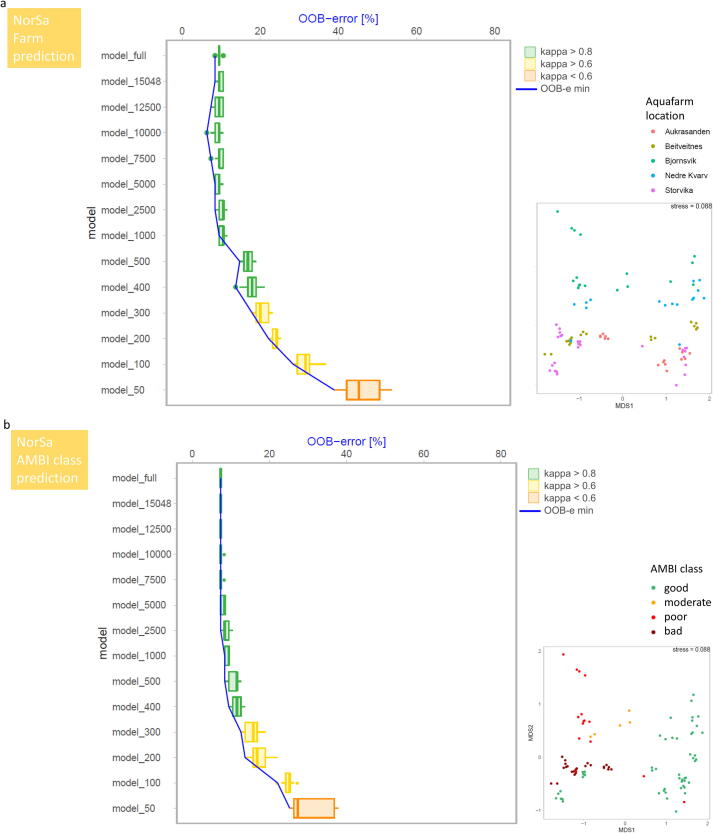
Change of OOB (out of bag error) and kappa value with decreasing dataset size (number of sequences) used for RF prediction for the NorSa dataset to predict the aquafarm location (a) and the AMBI ecological quality index based on benthic bacterial community composition. For further details see legend of Fig. 4.

NorSa (Fig. 7): For the NorSa dataset, we used the bacterial V3-V4 16S rDNA metabarcodes obtained from each of the 95 individual samples to predict two variables. First, the geographic location of the salmon farming site (Fig. 7a, predicted categories: Aukrasanden, Beitveitness, Bjornsvik, Nedre Kvarv and Storvika). Second, the biotic index AMBI (and resulting EQ category) for each sample, which was originally obtained from macroinvertebrates during a routine compliance monitoring of these salmon farms (predicted categories: good, moderate, poor, or bad ecological quality). Despite this dataset had the lowest sample saturation (coverage) (Fig. 3d), prediction accuracy for EQ category was similarly high for the full dataset (92.6%) and for the dataset that included only 1000 sequences per sample (90.8%) (Fig. 7b). Kappa statistics revealed a high agreement between actual and predicted EQ category for each of the 95 samples for the full dataset and for the model_1000 datasets (0.88 and 0.84, respectively). At 500 and 400 sequences per sample, the mean precision of prediction dropped to 89.0% and 88.1% respectively, yet kappa values were still > 0.8. Only when number of sequences decreased below 400 sequences per sample, kappa values decreased below this threshold.

Table 1 summarizes these results and provides a comparative overview of the average number of sequences in the full data of each dataset and the minimum sampling size at which RF predictions for the individual variables were still in the same (kappa and accuracy) category compared to the full dataset. This overview shows that in the “worst case scenario” (ScoSa – distance from cage prediction, Fig. 4a), as few as 5000 sequences per sample were required to achieve RF prediction accuracies as good as for the corresponding full dataset (with 37.642 sequences per sample on average).

**Table 1:** Summary of RF prediction results. The table shows the lower boundary (sequence numbers) at which an RF prediction performance was achieved in downsampled datasets that matched the prediction performance of the respective full dataset (=targeted RF prediction performance).

Data set	n samples*	Averaged n reads per sample in full dataset	RF reference label for prediction	n prediction classes	Min n sequences required for targeted RF prediction performance**
ScoSa	76	37,642	Station (distance)	3	5000
			Salmon production phase	3	50
BasCo	39	76,259	microgAMBI	4	5000
BallWa	59	91,598	Country of origin	3	2500
NorSa	95	31,057	Aquafarm location	5	1000
			AMBI	4	1000

*Numbers refer to sample numbers which were used in this study. These are samples which had at least 15,000 sequences, which was the minimum number that we chose as highest threshold for downsampled datasets.

**Full datasets as reference.

## 4 Discussion

Advances in various high-throughput sequencing technologies have opened up new opportunities to exploit massive sequence datasets from environmental samples for developing prognostic and predictive markers for biomonitoring. RF learning algorithms are increasingly used in microbial ecology for classification problems [31], [42]. While several studies have compared the power of RF classifications with other classifiers or different model parameters within RF, no environmental sequencing study has evaluated the relationship between training data volume and RF accuracy. Therefore, in the specific problem addressed in our study, we are asking, how many amplicon sequences of marine bacterial communities are required per sample to achieve a desired level of RF prediction performance. A common sense logic could be “the more data, the better”. However, the larger the (amplicon) datasets, the more expensive are the sequencing costs and the longer is the computation time needed to train the model. In addition, too much data (=too many features) may have a tendency to overfit (lower variation in individual trees resulting in a “less random” forest). This is prevented using smaller datasets (increase of variation in the individual trees within the forest, decorrelation) [40], [43]. However, removing too many features can impair model performance. Thus, finding the ideal number of sequences per sample is an important step towards an optimal sampling design for environmental sequencing studies which use RF learning algorithms for sample classification.

Our results from four test datasets (V3-V4 16S rDNA gene amplicon surveys of marine bacterial communities from different environments) showed that there is no general answer to this question. Depending on the dataset and the question asked, we identified subsets as little as 50 sequences as powerful as the full dataset of 15,000 sequences (ScoSa salmon production phase prediction) and in other cases the subsets required a minimum of 5000 sequences (ScoSa distance predictions, BasCo microgAMBI predictions) to achieve as accurate predictions as the full dataset.

The discrepancies could be explained by the complexity of the problem addressed in this study. Statistical heuristic to determine a suitable number of sequences per sample for classification problems are a function of the number of classes in the classification, the distribution of classes across the complete dataset (=balance), the total number of samples, and the ability of the training data to adequately characterize the classes being mapped [44]. Also, the tuning parameters when building a random forest could play a role in this context [45].

Here, we can largely exclude some of these factors as explanations for the observed discrepancies of 50 versus 5000 sequences per sample as minimal data size. The number of classes is relatively even among the test datasets of this study (min 3, max 5). In addition, the maximum discrepancy in the minimum dataset was observed within the same number of classes (n = 3, ScoSa salmon production phase prediction and ScoSa distance prediction). And, finally, the dataset with the highest number of classes (NorSa aquafarm location prediction) required fewer sequences per sample (n = 1000) for a full dataset-like classification than a dataset with the minimum number of classes (BallWa, min n sequences = 2500).

Likewise, the total number of samples in a dataset is not an obvious decisive factor that determines the minimum number of sequences required for the targeted level of RF performance. The highest number of samples (n = 95) was available for the NorSa dataset. However, the minimum number of sequences required for the targeted level of RF performance was notably higher compared to a dataset with only 76 samples (ScoSa salmon production phase prediction). Furthermore, in the ScoSa dataset, the same number of samples (n = 76) required only 50 sequences minimum to predict salmon production phase (3 classes), while 5000 min sequences were needed to predict distance to the salmon farming site (also 3 classes).

We also consider it unlikely that the tuning parameters when building a random forest could play a role for the obtained results. Relevant tuning parameters are the choice of the base tree, the number of trees in the forest, size of the leaf nodes and the rate of data subsampling [45]. We have built 6000 trees, which outnumbers the number of trees used in comparable analyses. For example, for the original analysis of the NorSa dataset, 300 trees were used [9]. 5000 trees were used for the original BallWa dataset analysis [31], and Smith et al. [28] used 1000 trees for RF analyses to classify unpolluted sites from those contaminated with uranium, nitrate, or oil using V4 16S rRNA gene amplicons of bacterial communities. Also, we have constructed ∼ 300 models with varying tuning parameters for each dataset. This strategy should minimize the effect of tuning parameters in the identification of the smallest number of sequences needed for the targeted classification performance.

In case of training data class imbalance, classifications may favor the classes that represent the largest proportion in the training samples (majority classes) [46]. Thus, classes that are under-represented in the training data may thus be difficult to classify correctly. It therefore seems reasonable to assume that the smaller the number of sequences become within under-represented classes, the more erroneous the classification performance. To test whether this helps to explain the different minimum sequence numbers that achieve targeted classification in the datasets analyzed in this study, we analyzed the (in)equality of class frequency distributions using the Gini coefficient (not to be confused with Gini impurity) and compared with the minimum number of sequences in a sample that is required for the targeted prediction performance. The results are presented in Supplementary File 3. They clearly show that a data class imbalance is irrelevant as factor to determine the required minimum of sequences within a sample for targeted prediction performance. Previous analyses showed that among several commonly used classifiers for “omics” data RF is the optimal choice when feature distributions are skewed and when class distributions are unbalanced [47].

We consider the ability of the training data to adequately characterize the classes being mapped as the best proxy to assess the number of sequences in a sample that is required for the targeted classification performance. Especially from RF classifications using image analyses, it is well known that classifications are more accurate, when classes are mutually exclusive and have hard, well-defined boundaries [44], [48]. A possibility to visualize how well the boundaries between classes are established based on the features observed for the samples within each of the class is ordination plots such as multidimensional scaling (MDS). The boundaries of classes (clusters in ordination plots) in such plots can be calculated and visualized using confidence intervals [49]. The smaller the overlap of confidence intervals of individual class-specific clusters, the higher is the probability that targeted RF classification accuracy can be achieved with a low number of sequences in a sample. Supplementary File 4 shows an example for the ScoSa dataset. While salmon production phase clusters are well separated with only little overlap of cluster-specific confidence intervals, distance clusters are less clearly separated. This corroborates well with our finding that in the former case only 50 sequences per sample are sufficient for the targeted RF classification accuracy, while in the latter case at least 5000 sequences are required. This observation confirms our assumption that datasets with well-defined boundaries of classes require fewer sequences within each sample in the training dataset to achieve the targeted classification performance. Subtle differences among the classes, such as environmental gradients, season or geographic origin will require more sequences within a sample. But even with very large sequence datasets, RF predictions may not be satisfying, if these sequences do not succeed to better define the boundaries of the classes. This was the case for the BasCo dataset analyzed in this study. Even when the full dataset (n = 76,259 sequences, sampled to near saturation) was used to train the RF model, the mean precision of prediction was only 78.3% at moderate agreement (kappa = 0.71) between reference EQ values and predicted EQ values.

Well-defined, hard boundaries between classes with little to no gradual transitions or edge overlap occur when the features within a class (here: bacterial V3-V4 16S rRNA gene amplicons) are as specific as possible for each individual class [44]. One way to visualize the class specificity of features is Venn diagrams. Not surprisingly, we found that the specificity of ASVs was notably higher for the ScoSa salmon production phase dataset (n min sequences required = 50) compared to the ScoSa distance dataset (n min sequences required = 5000) (Supplementary File 5). In the latter, 35% of all sequences were common to all three distance classes, whereas only 11% of all sequences were common to all three salmon production phases.

The coefficient of variation (CV) of each feature in a dataset can be interrogated as a measure to assess the ability of features (here: ASVs) to discriminate prediction classes. The CV measures the standard deviation of an individual feature across the individual prediction classes relative to the CV group mean (“group” describes the prediction target) [50]. The general expectation is as follows: the higher the CV of an individual feature (ASV), the more specific is its occurrence in individual prediction classes (=uneven distribution of an ASV, which leads to a higher CV). In conclusion, the more features with a higher CV are included in a dataset, the higher is the likelihood to obtain a more accurate RF prediction with only a subset of the features included in a dataset. This is because the subset with fewer features still includes sufficient information in each feature for reliable RF predictions. To test this logic, we have exemplary calculated the CV for the ScoSa salmon production phase dataset and for the ScoSa station dataset. Indeed, the ScoSa salmon production phase dataset, which has still reliable RF prediction accuracies with as few as 50 features, has a notably higher CV density distribution compared to the ScoSa station dataset, which requires at least 5000 features to achieve the targeted RF prediction performance. For CV kernel density plots and more detailed information and analyses we refer to Supplementary File 6.

## 5 Conclusions

In conclusion of our study, even for the “worst case scenario” when classes had no hard boundaries but substantial gradual transition and edge overlap (Supplementary File 4) we identified 5000 sequences as a threshold for the number of sequences within a sample, beyond which no substantial improvements are achieved in RF classification performance. This could be a rule of thumb guiding future studies using taxonomic metabarcodes of marine microbial communities for RF classification in ecological studies. Our examples included classifications of environmental quality and stressor impact, as well as spatial and temporal scaling, all of which are central topics in microbial ecology. Considering that environmental DNA metabarcoding studies of marine microbial communities usually acquire substantially higher number of sequences [9], [17], [31], [36], [37], [51] without prior adaptations of sequencing depths to the research questions addressed, our study may guide future sampling designs in RF classification based on microbial amplicon sequences to save financial and computational resources, while avoiding possible bias of overfitting and reducing noise due to too large datasets. Also, our study has identified parameters that are helpful to assess whether fewer or more sequences are needed as features to distinguish prediction classes. Both, feature specificities as well as multidimensional scaling plots allow for assessments of the minimum sequencing depth required for an RF performance that does not improve substantially with notably larger sequencing efforts. We therefore recommend a small-scale pilot study before designing large-scale experiments to assess the general tendency of features (sequences within each sample) to distinguish prediction classes.

## CRediT authorship contribution statement

**Verena Dully:** Writing - review & editing. **Thomas A. Wilding:** Writing - review & editing. **Timo Mühlhaus:** Writing - review & editing. **Thorsten Stoeck:** Supervision, Conceptualization, Funding acquisition, Writing - original draft.

## Declaration of Competing Interest

The authors declare that they have no known competing financial interests or personal relationships that could have appeared to influence the work reported in this paper.
