# Supplementary material for: Identifying the minimum amplicon sequence depth to adequately predict classes in eDNA-based marine biomonitoring using supervised machine learning
Source: Comput Struct Biotechnol J. 2021 Apr 26;19:2256–68. doi: 10.1016/j.csbj.2021.04.005 (PMC8093828; doi:10.1016/j.csbj.2021.04.005)
Supplement: Supplementary Data 2 [file mmc2.docx]

*Supplementary file 2:* This supplemental document is intended to clarify how random forest (RF) models were constructed during the analysis. The novel ScoSa data set was used as an example. In the first step (Step 1), full models (FM) are constructed. For this RF models, the complete ScoSa dataset containing all available sequences was used. In the second step (Step 2), the RF models are built on a reduced, down-sampled ScoSa dataset.

**STEP 1**

1a.) A full RF model is constructed using all available sequences of the dataset (on average

37,642 sequences per sample). For this FM, mtry is set to default value (here:

mtry=55). This results in one model for step 1a.

1b.) Using the same dataset with mtry set to:

default value +1 (mtry=56),

default value +2 (mtry=57),

default value +3 (mtry=58).

This results in tree models for step 1b.

1c.) Using the same dataset with mtry set to:

default value -1 (mtry=54),

default value -2 (mtry=53),

default value -3 (mtry=52).

This results in tree models for step 1c.

1d.) Repeat the 7 models from step 1a-1c two times. This results in 21 RF models for step 1 in total.

**STEP 2**

2a.) The dataset is downsampled to the minimum sequence number per sample (here:

15,177 sequences). This step is conducted using the rrarefy function.

2b.) Using this rarefied dataset for new RF models as described in 1b-1d.

This results in 21 models for step 2b.

2c.) Deeper rarefaction to given sequence numbers. The dataset is downsampled to

12 different sequence numbers per sample: 12,500, 10,000, 7500, 5000, 2500, 1000, 500, 400, 300, 200, 100 and 50 sequences.

Using these 12 rarefied data sets for new RF models as described in 1a, 1b and 1c with seven models each. This results in 12*7 = 84 models for step 2c.

2d.) All of the 84 models from step 2c are repeated two times using different base

trees. This results in 168 models for step 2d. This results in 273 RF models for step 2 in total.
