# Supplementary material for: Identifying the minimum amplicon sequence depth to adequately predict classes in eDNA-based marine biomonitoring using supervised machine learning
Source: Comput Struct Biotechnol J. 2021 Apr 26;19:2256–68. doi: 10.1016/j.csbj.2021.04.005 (PMC8093828; doi:10.1016/j.csbj.2021.04.005)
Supplement: Supplementary Data 3 [file mmc3.pptx]

## Slide 1
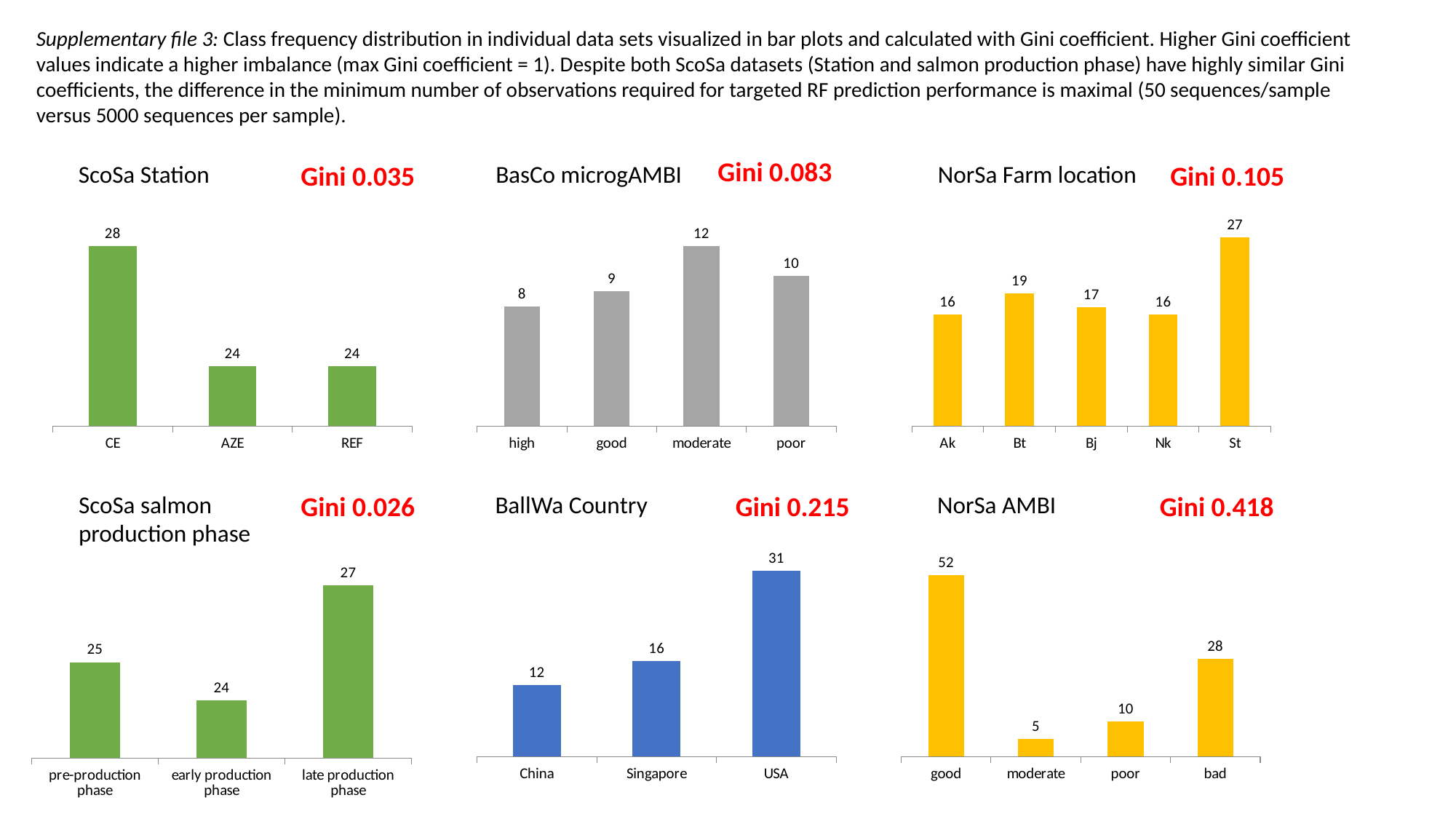

Supplementary file 3: Class frequency distribution in individual data sets visualized in bar plots and calculated with Gini coefficient. Higher Gini coefficient values indicate a higher imbalance (max Gini coefficient = 1). Despite both ScoSa datasets (Station and salmon production phase) have highly similar Gini coefficients, the difference in the minimum number of observations required for targeted RF prediction performance is maximal (50 sequences/sample versus 5000 sequences per sample).
Gini 0.083
ScoSa Station
Gini 0.035
BasCo microgAMBI
NorSa Farm location
Gini 0.105
### Chart
| Category | |
|---|---|
| CE | 28.0 |
| AZE | 24.0 |
| REF | 24.0 |
### Chart
| Category | |
|---|---|
| high | 8.0 |
| good | 9.0 |
| moderate | 12.0 |
| poor | 10.0 |
### Chart
| Category | |
|---|---|
| Ak | 16.0 |
| Bt | 19.0 |
| Bj | 17.0 |
| Nk | 16.0 |
| St | 27.0 |ScoSa salmon
production phase
Gini 0.026
BallWa Country
Gini 0.215
NorSa AMBI
Gini 0.418
### Chart
| Category | |
|---|---|
| China | 12.0 |
| Singapore | 16.0 |
| USA | 31.0 |
### Chart
| Category | |
|---|---|
| good | 52.0 |
| moderate | 5.0 |
| poor | 10.0 |
| bad | 28.0 |
### Chart
| Category | |
|---|---|
| pre-production phase | 25.0 |
| early production phase | 24.0 |
| late production phase | 27.0 |

## Slide 2
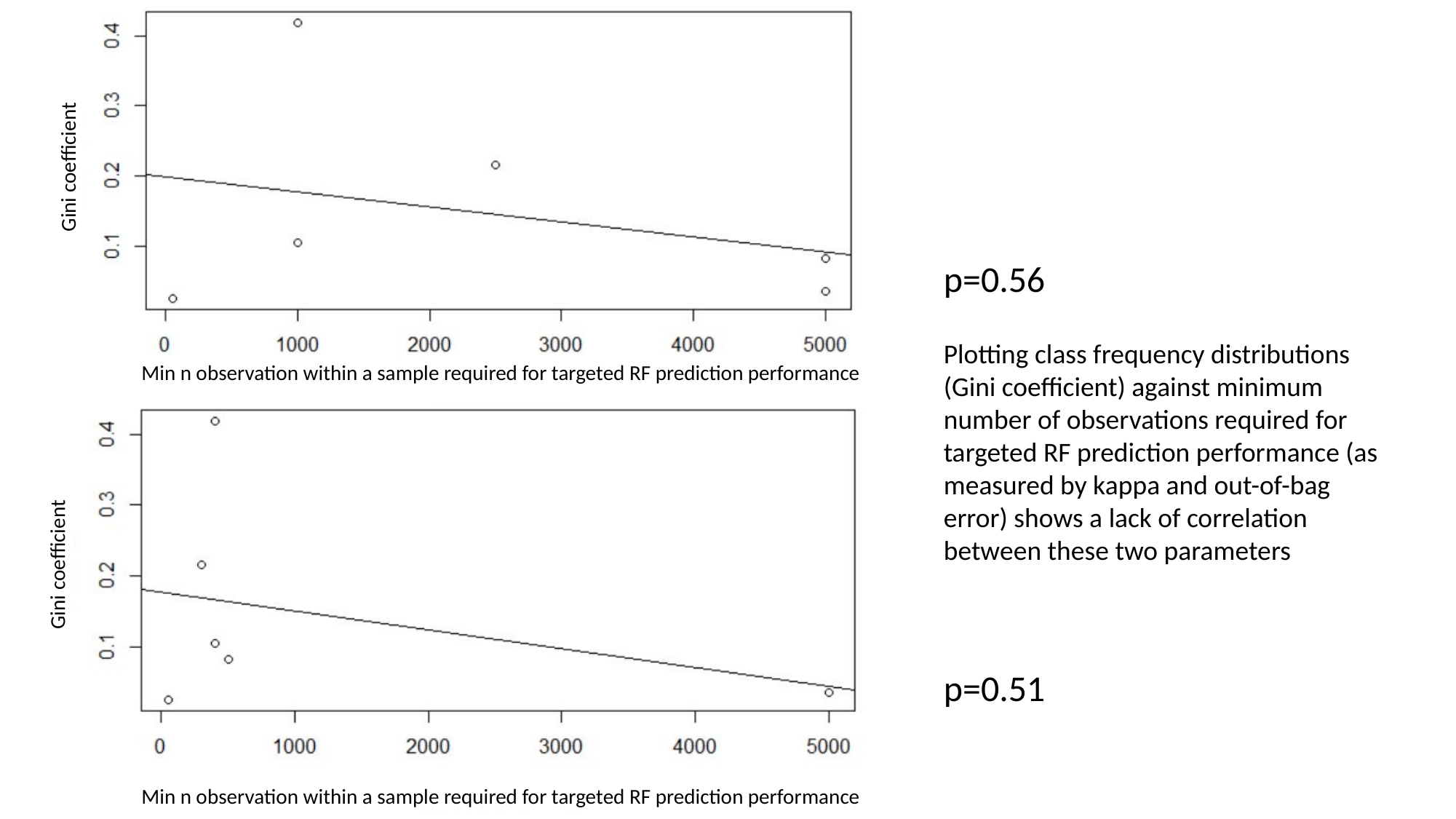

Gini coefficient
p=0.56
Plotting class frequency distributions (Gini coefficient) against minimum number of observations required for targeted RF prediction performance (as measured by kappa and out-of-bag error) shows a lack of correlation between these two parameters
Min n observation within a sample required for targeted RF prediction performance
Gini coefficient
p=0.51
Min n observation within a sample required for targeted RF prediction performance
