# Supplementary material for: Identifying the minimum amplicon sequence depth to adequately predict classes in eDNA-based marine biomonitoring using supervised machine learning
Source: Comput Struct Biotechnol J. 2021 Apr 26;19:2256–68. doi: 10.1016/j.csbj.2021.04.005 (PMC8093828; doi:10.1016/j.csbj.2021.04.005)
Supplement: Supplementary Data 4 [file mmc4.pptx]

## Slide 1
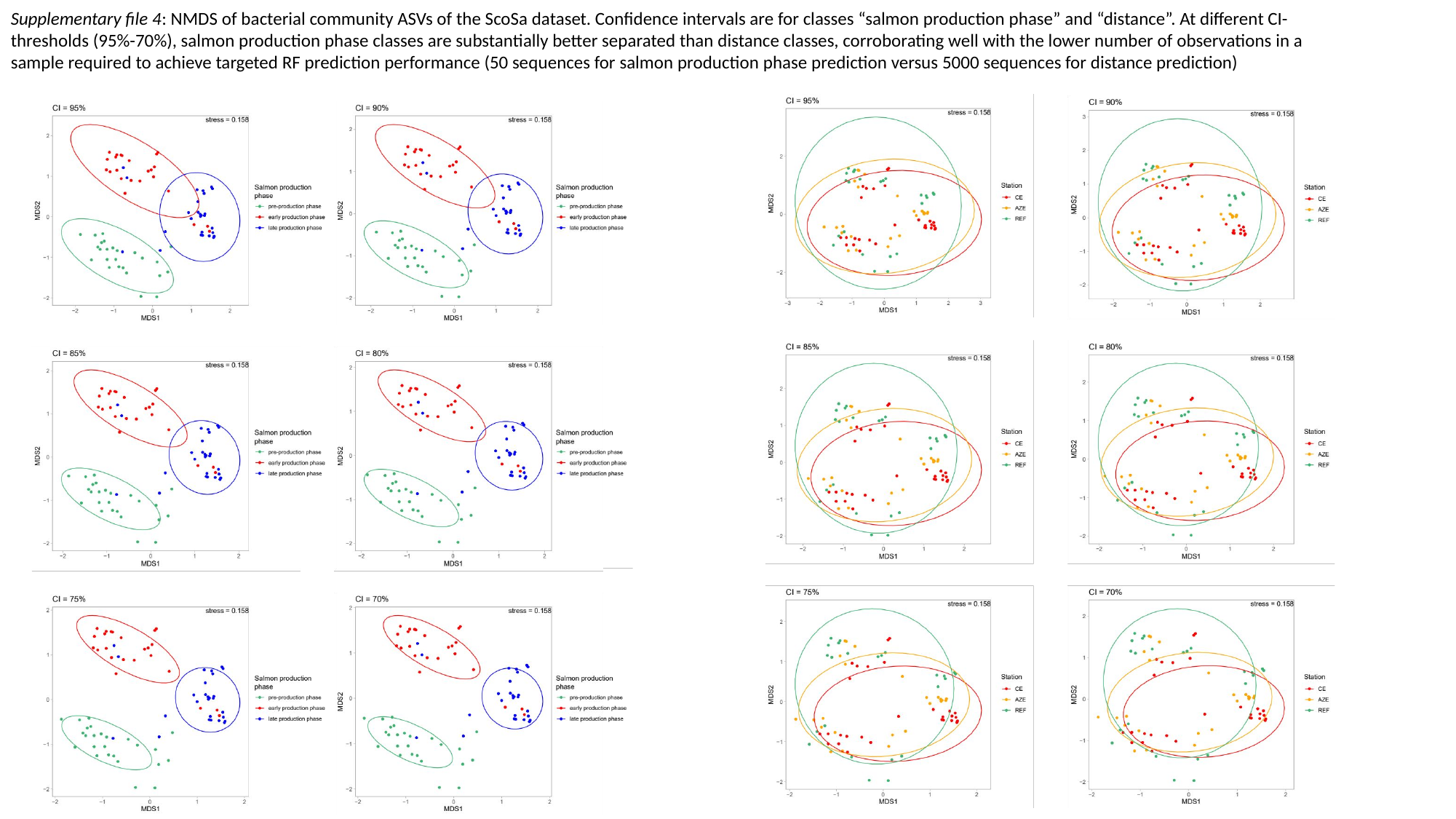

Supplementary file 4: NMDS of bacterial community ASVs of the ScoSa dataset. Confidence intervals are for classes “salmon production phase” and “distance”. At different CI-thresholds (95%-70%), salmon production phase classes are substantially better separated than distance classes, corroborating well with the lower number of observations in a sample required to achieve targeted RF prediction performance (50 sequences for salmon production phase prediction versus 5000 sequences for distance prediction)
