# Supplementary material for: Identifying the minimum amplicon sequence depth to adequately predict classes in eDNA-based marine biomonitoring using supervised machine learning
Source: Comput Struct Biotechnol J. 2021 Apr 26;19:2256–68. doi: 10.1016/j.csbj.2021.04.005 (PMC8093828; doi:10.1016/j.csbj.2021.04.005)
Supplement: Supplementary Data 5 [file mmc5.pptx]

## Slide 1
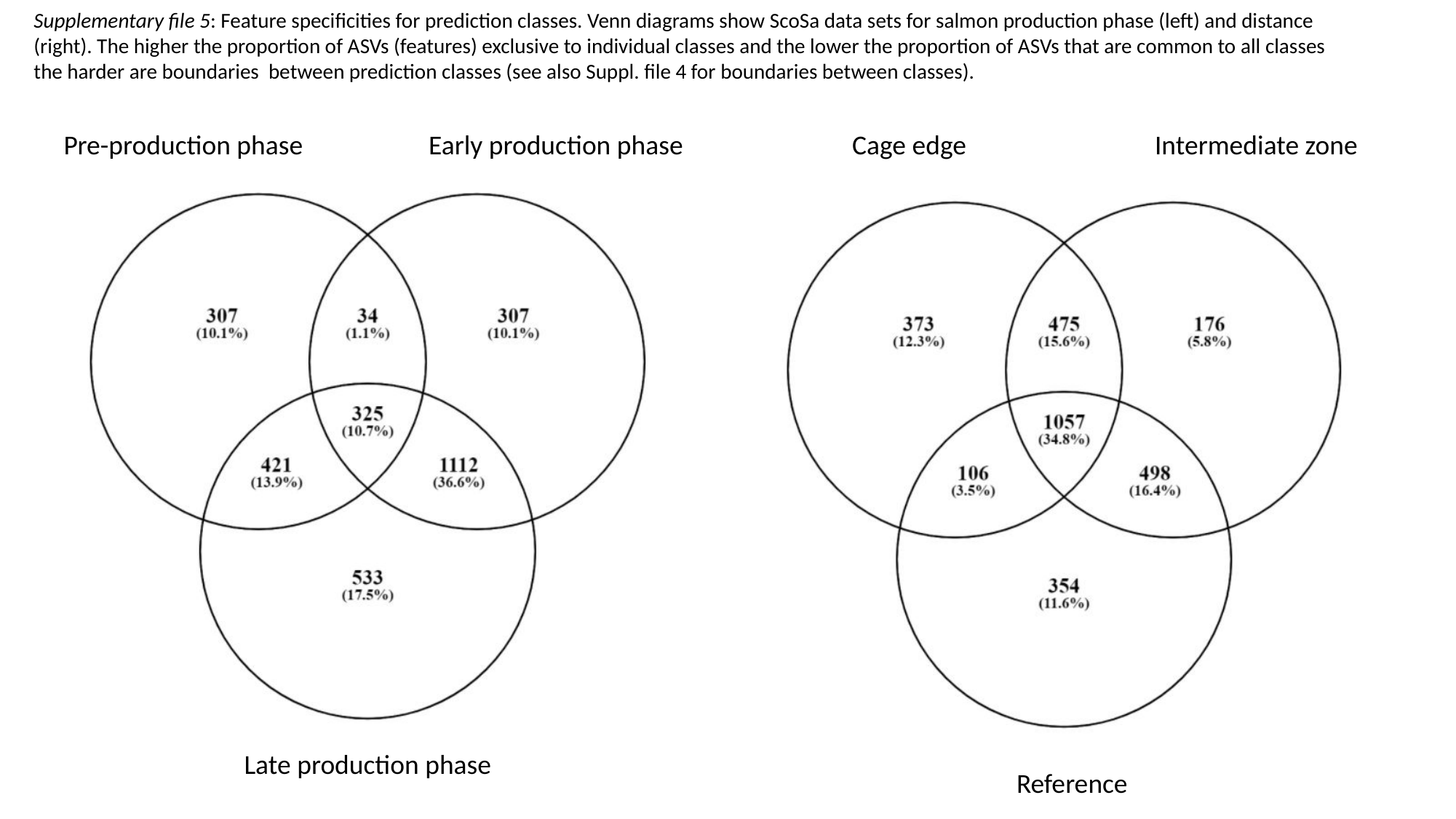

Supplementary file 5: Feature specificities for prediction classes. Venn diagrams show ScoSa data sets for salmon production phase (left) and distance (right). The higher the proportion of ASVs (features) exclusive to individual classes and the lower the proportion of ASVs that are common to all classes the harder are boundaries between prediction classes (see also Suppl. file 4 for boundaries between classes).
Pre-production phase
Early production phase
Summer
Late production phase
Autumn
Cage edge
Intermediate zone
Reference
