# Supplementary material for: Identifying the minimum amplicon sequence depth to adequately predict classes in eDNA-based marine biomonitoring using supervised machine learning
Source: Comput Struct Biotechnol J. 2021 Apr 26;19:2256–68. doi: 10.1016/j.csbj.2021.04.005 (PMC8093828; doi:10.1016/j.csbj.2021.04.005)
Supplement: Supplementary Data 6 [file mmc6.pptx]

## Slide 1
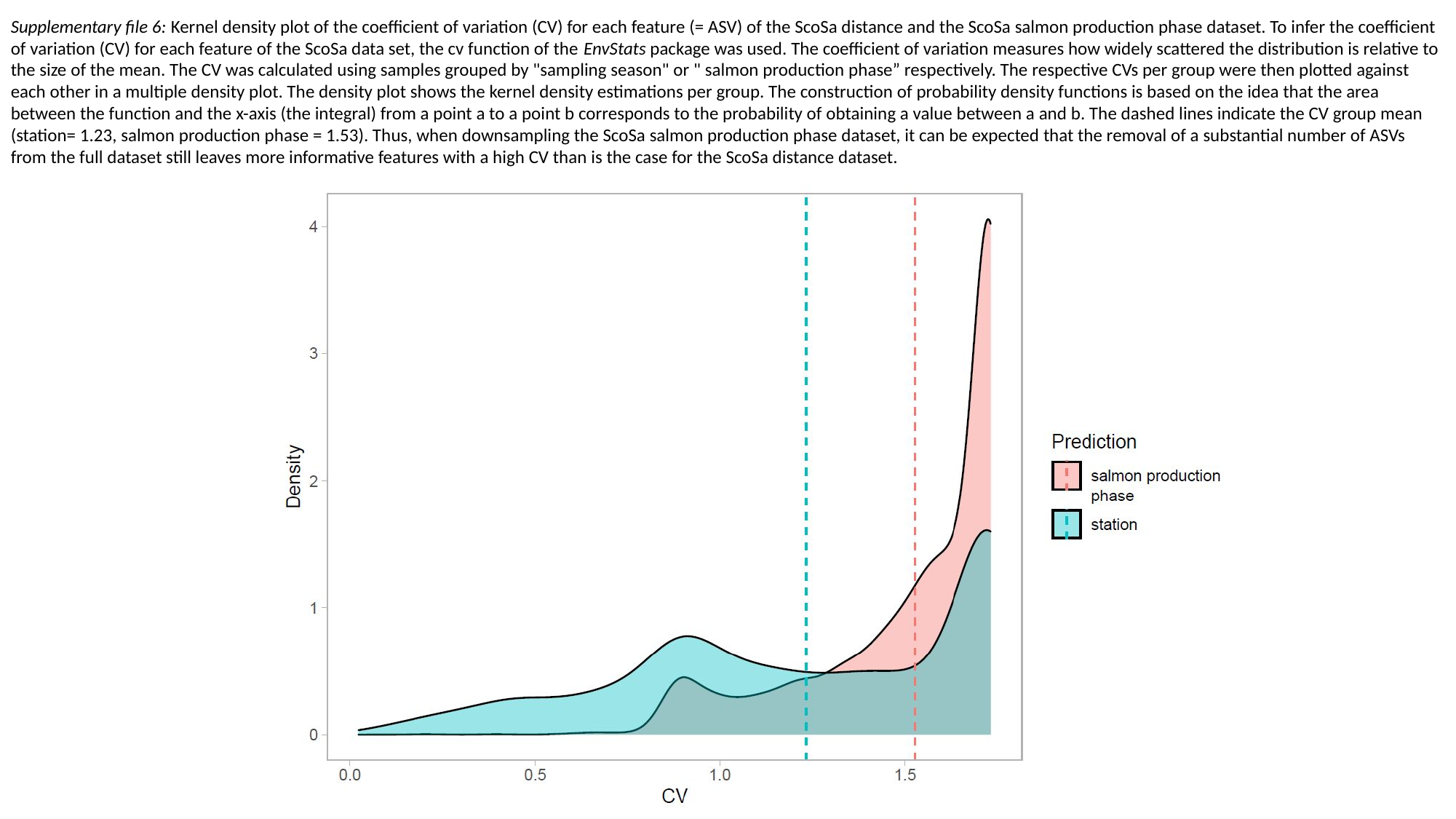

Supplementary file 6: Kernel density plot of the coefficient of variation (CV) for each feature (= ASV) of the ScoSa distance and the ScoSa salmon production phase dataset. To infer the coefficient of variation (CV) for each feature of the ScoSa data set, the cv function of the EnvStats package was used. The coefficient of variation measures how widely scattered the distribution is relative to the size of the mean. The CV was calculated using samples grouped by "sampling season" or " salmon production phase” respectively. The respective CVs per group were then plotted against each other in a multiple density plot. The density plot shows the kernel density estimations per group. The construction of probability density functions is based on the idea that the area between the function and the x-axis (the integral) from a point a to a point b corresponds to the probability of obtaining a value between a and b. The dashed lines indicate the CV group mean (station= 1.23, salmon production phase = 1.53). Thus, when downsampling the ScoSa salmon production phase dataset, it can be expected that the removal of a substantial number of ASVs from the full dataset still leaves more informative features with a high CV than is the case for the ScoSa distance dataset.
